# *Abelmoschus esculentus* Ameliorates Cognitive Impairment in Hyperlipidemic ApoE^−/−^ Mice via Modulation of Oxidative Stress and Neuronal Differentiation

**DOI:** 10.3390/antiox14080955

**Published:** 2025-08-04

**Authors:** Chiung-Huei Peng, Hsin-Wen Liang, Chau-Jong Wang, Chien-Ning Huang, Huei-Jane Lee

**Affiliations:** 1 Division of Basic Medical Science, Hungkuang University, No. 1018, Section 6, Taiwan Boulevard, Shalu District, Taichung City 43302, Taiwan; a222907@sunrise.hk.edu.tw; 2 Department of Internal Medicine, Chung Shan Medical University Hospital, No. 110, Section 1, Jianguo N. Road., South District, Taichung City 40201, Taiwan; 3 Department of Medical Research, Chung Shan Medical University Hospital, Taichung 40201, Taiwan; wcj@csmu.edu.tw; 4 Department of Health Industry Technology Management, Chung Shan Medical University, Taichung 40201, Taiwan; 5 Institute of Medicine, Chung-Shan Medical University, Taichung City 40201, Taiwan; 6 Department of Biochemistry, School of Medicine, Chung Shan Medical University, No. 110, Section 1, Jianguo N. Road., South District, Taichung City 40201, Taiwan; 7 Department of Clinical Laboratory, Chung Shan Medical University Hospital, Taichung City 40201, Taiwan

**Keywords:** high-fat diet, ApoE^−/−^ mice, cardiovascular disease, hyperlipidemia, cognitive impairment, Abelmoschus esculentus

## Abstract

Cardiovascular disease (CVD) and dementia may share common pathogenic factors such as atherosclerosis and hyperlipoproteinemia. Dyslipidemia-induced oxidative stress contributes to dementia comorbidity in CVD. *Abelmoschus esculentus* (AE, okra) potentiates in alleviating hyperlipidemia and diabetes-related cognitive impairment. This study evaluated the effects of AE in hyperlipidemic ApoE^−/−^ mice treated with streptozotocin (50 mg/kg) and fed a high-fat diet (17% lard oil, 1.2% cholesterol). AE fractions F1 or F2 (0.65 mg/kg) were administered for 8 weeks. AE significantly reduced serum LDL-C, HDL-C, triglycerides, and glucose, improved cognitive and memory function, and protected hippocampal neurons. AE also lowered oxidative stress markers (8-hydroxy-2′-deoxyguanosine, 8-OHdG) and modulated neuronal nuclei (NeuN) and doublecortin (DCX) expression. In vitro, AE promoted neurite outgrowth and neuronal differentiation in retinoic acid (RA)-differentiated human SH-SY5Y cells under metabolic stress (glucose and palmitate), alongside the upregulation of heme oxygenase-1 (HO-1), Nuclear factor-erythroid 2-related factor 2 (Nrf2), and brain-derived neurotrophic factor (BDNF). These findings suggest AE may counter cognitive decline via oxidative stress regulation and the enhancement of neuronal differentiation.

## 1. Introduction

Cognitive disorders represent the initial stage of dementia, a progressive and debilitating condition that is among the most resource-intensive geriatric diseases. Currently, dementia affects over 55 million individuals worldwide, and projections estimate an increase to 139 million cases within the next 25 years [1]. The hallmark symptoms of dementia include impairments in memory, language, problem-solving, and other cognitive functions. Alzheimer’s disease (AD) is the most prevalent form of dementia, with diagnosis often based on the presence of toxic β-amyloid deposits. Vascular dementia is the second most common form and is distinguished from AD by evidence of prior cerebrovascular events, such as stroke or hemorrhage, and the presence of ongoing cerebrovascular disease [2]. Subclinical cardiovascular disease (CVD) such as hypertension, intra- and extracranial atherosclerosis, and arteriosclerosis in midlife may contribute to the later development of dementia, including AD [3]. Emerging evidence suggests a significant association between CVD and approximately 25% of dementia [4,5]. The common pathophysiological mechanisms include chronic inflammation and oxidative stress [6]. Given these associations, further research is warranted to elucidate the relationship between CVD and cognitive disorders and to explore potential preventive and therapeutic strategies.

CVD and dementia may share common genetic factors, including mutations in the presenilin and apolipoprotein E (ApoE) genes [7]. ApoE is involved in the lipid metabolism and conversion of lipoproteins [8] and is a critical factor in various cardiovascular and neurodegenerative conditions, including cerebral infarction and dyslipidemia. Dyslipidemia can increase oxidative stress and result in elevated reactive oxygen species (ROS). Vascular dementia has been associated with ROS-induced damage at multiple levels of biological organization [9]. These findings underscore the interconnectedness of lipid metabolism, oxidative stress, and inflammation in CVD and dementia, thereby highlighting the necessity of further investigating shared mechanistic pathways and potential therapeutic interventions.

Apolipoprotein E knockout (ApoE^−/−^) mice are among the most widely utilized animal models for CVD research [10] as they spontaneously develop hypercholesterolemia and arterial lesions within a few weeks after birth [11,12]. ApoE plays a crucial role in cholesterol transport between glial cells and neurons and is the primary apolipoprotein secreted in the brain [13]. In ApoE^−/−^ mice, oxidative stress and inflammation may contribute to an increased risk of neurodegeneration, with dendritic neurite alterations observed as early as four months of age [14]. Additionally, decreased neuronal excitability has been reported to lead to cognitive impairment in this model [15]. Although ApoE^−/−^ mice are characterized by dyslipidemia, numerous studies have indicated that a high-fat diet (HFD) exacerbates metabolic disturbances, thereby accelerating the pathological processes observed in this model. Compared to ApoE^−/−^ mice maintained on a standard diet, those fed an HFD exhibit significantly increased inflammatory markers, higher plaque burden, elevated lipid accumulation, and an increased spontaneous rupture rate in the brachiocephalic and coronary arteries [16]. Recently, an alternative animal model was developed in which ApoE^−/−^ mice were subjected to an HFD to induce aortic atherosclerosis, followed by an injection of streptozotocin (STZ) (130 mg/kg) to induce hyperglycemia. In this model, the formation of atherosclerotic lesions in the thoracic and abdominal aorta is more pronounced compared to mice receiving only an HFD [17,18]. Furthermore, hyperglycemia, dyslipidemia, and systemic inflammation are significantly elevated in these mice relative to those fed only an HFD [18]. However, cognitive impairment and protein abnormalities in neural tissues have been less frequently reported in this model. Additionally, further research is required to determine whether preventive interventions targeting neurodegeneration are effective in this context.

*Abelmoschus esculentus* (AE, okra) is a fruit widely utilized in traditional medicine for its beneficial effects on metabolic syndrome [19]. Several AE fractions have been isolated through sequential extraction, F1 (rich in quercetin glucosides and triterpene esters) and F2 (containing carbohydrates and polysaccharides), identified as particularly effective in attenuating hyperglycemia and improving lipid profiles [20]. Quercetin glycosides have been reported to modulate gut microbiota composition and may contribute to CVD prevention [21]. Emerging evidence also suggests that these compounds may enhance cognitive function in the elderly [22]. In addition, triterpene esters derived from natural products exhibit notable bioactivities, including antioxidant properties, the inhibition of acetylcholinesterase and butyrylcholinesterase, and antimicrobial effects [23,24,25]. Polysaccharides, as natural high-molecular-weight polymers, possess a broad range of biological activities, such as the regulation of intestinal microbiota, the amelioration of glucose and lipid metabolic disorders, and antioxidative functions. Owing to their physiological versatility, plant-derived polysaccharides are increasingly being explored for applications in clinical therapies and functional food development. F2, enriched polysaccharides, appears to exert its effects, at least partially, by alleviating gut microbiota dysbiosis. These findings suggest that AE holds promise as a potential therapeutic agent for both metabolic disorders and neurodegenerative diseases [26,27]. Specifically, in in vitro studies, AE reduced neuropathological hallmarks of Alzheimer’s disease by preventing neuronal damage induced by beta-amyloid (A*β*) [28] and, conversely, attenuated palmitate (PA)-induced A*β* production [29]. In a type 2 diabetes animal model, AE was found to enhance neuronal autophagy, thereby preventing hippocampal damage and improving cognitive and emotional function [30]. Furthermore, recent findings indicate that AE improves hippocampal function in HFD-fed db/db mice and may promote neurogenesis [26].

The present study aims to investigate whether AE fractions, F1 and F2, attenuate oxidative stress, promote neuronal differentiation, and consequently improve cognitive function. For the in vivo experiments, ApoE^−/−^ mice were utilized to induce metabolic disturbances through an HFD and STZ administration. To assess neuronal development, doublecortin (DCX) was used as an early neuroblast marker during embryogenesis, while the neuronal nuclei (NeuN) antigen was analyzed as a marker of mature neurons during subsequent migration and differentiation [31]. For the in vitro studies, an all-trans-retinoic acid (RA)-induced model of neuronal differentiation was employed. Previous studies have reported that RA effectively increases neurite number and length, sequentially promoting neuronal migration and differentiation [31]. Therefore, microtubule-associated protein 2 (MAP2) and glial fibrillary acidic protein (GFAP) were used as markers to assess neurite growth. Furthermore, in the later stages of neurogenesis, brain-derived neurotrophic factor (BDNF) and its receptor, tropomyosin-related kinase B (TrkB), play a crucial role in neuroblast migration [32].

## 2. Materials and Methods

### 2.1. AE Preparation

AE was obtained from Chuchi, Chiayi, Taiwan. Fractions F1 and F2 were prepared according to established procedures (Figure S1) [33]. Briefly, fresh AE was mechanically crushed, extracted with 95% ethanol, and centrifuged. The resulting supernatant, designated F1 (1.08% yield), was analyzed by LC-MS/MS and found to contain quercetin glucoside (4.901 mg/g dry weight (DW)) and triterpene ester (4.301 mg/g DW) (Figure S2 and Table S1). The remaining residue from the initial ethanol extraction was subsequently extracted with deionized water and centrifuged to yield the water-extracted fraction, F2 (12.59% yield). A gel permeation chromatography analysis of F2 indicated an estimated molecular weight of 671 kDa. A compositional analysis of F2 revealed the presence of uronic acid (23.14%), galactose (18.92%), glucose (18.26%), and myoinositol (14.21%) and trace amounts of rhamnose, fucose, and glucosamine (Table S2).

### 2.2. Animals and Experimental Design

**Figure 1:**
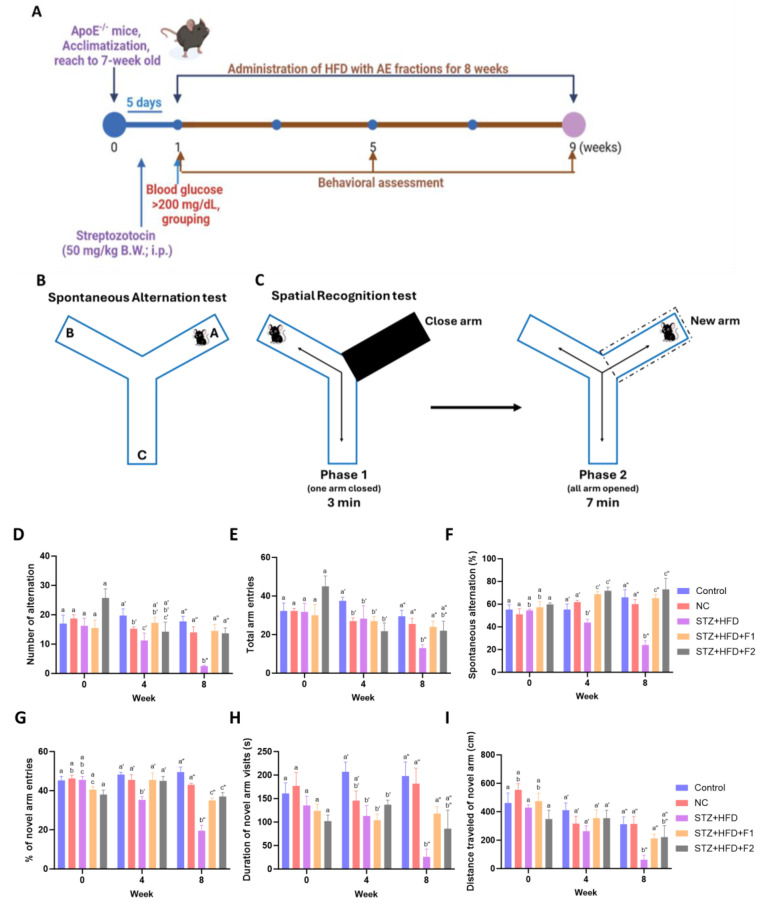
The effect of AE fractions on cognitive impairment. ApoE^−/−^ mice were treated with intraperitoneal-injected STZ at a dose of 50 mg/kg body weight and an HFD and subsequently supplemented with 0.65 mg/kg body weight of F1 or F2 for 8 weeks. The control and NC (ApoE^−/−^ mice) groups were given the control diet. (**A**) Timeline in the experiment; analysis for (**B**) spontaneous alternation; and (**C**) spatial recognition. Cognitive performance was assessed by analyzing (**D**) the number of alternations; (**E**) total arm entries; and (**F**) the percentage of spontaneous alternation in the Y-maze. Spatial recognition learning was assessed by measuring (**G**) the percentage of novel arm entries; (**H**) the duration of novel arm visits; and (**I**) the distance traveled of the novel arm. Values are represented as mean ± SE; values not sharing a common letter are significantly different (*p* < 0.05).

Male C57BL/6J wild-type mice and C57BL/6-Apoe^em1Narl^/Narl (ApoE^−/−^) mice, aged 4–5 weeks, were obtained from the National Center for Biomodels (NCB, Taipei, Taiwan). Animals were utilized for this research upon the completion of the acclimation period and when they reached seven weeks of age. The animals were housed under stable conditions, including a constant temperature of 22  ±  2 °C, a humidity of 55  ±  5%, and a 12 h light/dark cycle, with free access to water. All experimental procedures were approved by the Institutional Animal Care and Use Committee (IACUC) of Chung Shan Medical University (approval no. 2838, on 13 February 2023). A modified protocol was applied to induce cardiovascular disease (CVD) (Figure 1A). According to the conditions established in the preliminary experiments (Table S3 and Figure S3), ApoE^−/−^ mice was intraperitoneal-injected with or without streptozotocin (STZ) at a dose of 50 mg/kg body weight for five consecutive days. One week post injection, mice exhibiting fasting plasma glucose levels exceeding 200 mg/dL were considered diabetic and included in the subsequent experimental procedures. Mice were then randomly assigned to one of five groups: (1) Control (C57BL/6J mice fed a standard chow diet, n = 4); (2) Negative Control (NC, ApoE^−/−^ mice fed a standard chow diet, n = 4); (3) STZ + HFD (STZ-induced ApoE^−/−^ mice fed a high-fat diet (HFD) composed of 17% lard oil (I-MEI Foods Co., Ltd., Taipei, Taiwan), 1.2% cholesterol (Sigma-Aldrich, St. Louis, MO, USA), and 0.2% sodium cholate hydrate (Sigma-Aldrich, St. Louis, MO, USA), n = 4); (4) STZ + HFD + F1 (STZ-induced ApoE^−/−^ mice fed the HFD and administered F1 at 0.65 mg/kg body weight/day via oral gavage, n = 4); and (5) STZ + HFD + F2 (STZ-induced ApoE^−/−^ mice fed the HFD and administered F2 at 0.65 mg/kg body weight/day via oral gavage, n = 3). Fresh diets were provided daily, and any remaining food was removed and weighed to determine daily food consumption throughout the eight-week experimental period. The dosage of F1 and F2 was based on previously established protocols [20,26]. Daily food intake was monitored and quantified throughout the eight-week experimental period. All experimental procedures were conducted in compliance with the Taiwan Animal Protection Act (Law No. 10500042801, enacted on 4 November 1998 and amended on 18 May 2016) and the animal care and management guidelines established by the Research and Development Department in Chung Shan Medical University (20 June 2014).

### 2.3. Behavioral Experiments

To assess working memory and exploratory activity, a Y-maze apparatus was used, consisting of three arms (length: 30 cm, width: 7 cm, and wall height: 15 cm) positioned at 120° angles to each other. Behavioral assessments were conducted at weeks 0, 4, and 8 (Figure 1B,C). Mice were placed at the central junction of the Y-maze to track movement patterns and arm entry distances. Behavioral activity was recorded for 10 min using a video camera [34]. The percentage of spontaneous alternation was calculated using the formula [number of alternations/(total arm entries − 2)] × 100%.

A spatial recognition test was conducted to evaluate spatial discrimination ability. Mice were initially familiarized with the Y-maze for 3 min with one arm blocked. Subsequently, all arms were opened, and the mice were allowed to explore for an additional 7 min [35]. Spatial recognition was analyzed by calculating the percentage of novel arm entries according to the following formula: [(number of new arm entries/(total arm entries − 1)] × 100%.

### 2.4. Blood Biochemical Analysis

Blood samples were collected via cardiac puncture at the time of sacrifice and centrifuged at 3000× *g* for 10 min. Levels of glucose, cholesterol, triglycerides (TGs), high-density lipoprotein cholesterol (HDL-C), and low-density lipoprotein cholesterol (LDL-C) were measured using enzymatic colorimetric assays and commercial kits (Randox Laboratories, Antrim, UK). Following the manufacturer’s instructions, glycated hemoglobin (HbA1c) levels were determined using a Fortress Diagnostics (Fortress Diagnostics Limited, Antrim, UK) assay kit.

### 2.5. Histological Examination

Brain specimens were dissected to isolate the hippocampus and fixed in 10% neutral buffered formalin for subsequent hematoxylin and eosin (H&E) and Nissl staining. For H&E staining, hippocampal sections were stained with hematoxylin to visualize cell nuclei, followed by counterstaining with eosin. For Nissl staining, sections were immersed in 0.1% cresyl violet solution (Abcam, Cambridge, UK) for 8 min. After rinsing with distilled water, the sections were dehydrated, mounted with coverslips, and examined under a TissueFAXS PLUS tissue cytometer (TissueGnostics, Vienna, Austria).

### 2.6. Immunofluorescence (IF) Examination

Paraffin-embedded hippocampal tissue sections (5 μm) were deparaffinized, treated with 3% H_2_O_2_ in methanol for 10 min, and subjected to antigen retrieval using 0.01 M citrate buffer (pH 6; Sigma, St. Louis, MO, USA) at 95 °C for 10 min. After cooling to 50 °C and washing twice with phosphate-buffered saline (PBS), slides were blotted dry and immunostained (BioTnA, Inc. Kaohsiung, Taiwan). Briefly, sections were blocked for 1 h at room temperature, washed twice with PBS, and incubated overnight at 4 °C with primary antibodies (anti-DCX, Cat. No. ab207175; anti-NeuN, Cat. No. ab177487; anti-8-hydroxy-2′-deoxyguanosine (8-OHdG), Cat. No. ab48508, from Abcam Co., Cambridge, UK) diluted 1:200. Following two washes with phosphate-buffered saline (PBS)-Tris, slides were incubated in the dark for 1 h at room temperature with secondary antibodies (anti-rabbit IgG-488, A-11008, or anti-mouse IgG-594, A-11005, from Invitrogen, Carlsbad, CA, USA) and diluted according to the manufacturer’s instructions. After two additional 5 min PBS washes, 4′,6-diamidino-2-phenylindole (DAPI) (Applied Biological Materials Inc., Richmond, BC, Canada) was applied for nuclear staining. Slides were mounted and imaged using a TissueFAXS PLUS tissue cytometer (TissueGnostics, Vienna, Austria).

### 2.7. Enzyme-Linked Immunosorbent Assay (ELISA)

Serum BDNF concentrations were determined using an ELISA kit (Abcam, Cambridge, UK) [36]. Mouse serum samples were diluted 1:10, and 50 μL of each diluted sample or standard solution was added to the wells of a 96-well microplate. An equal volume (50 μL) of antibody-containing reagent was then added to each well. The plate was incubated at 400 rpm for 1 h at room temperature. After incubation, wells were washed three times with 350 μL of wash buffer. Subsequently, 100 μL of TMB (3,3′,5,5′-tetramethylbenzidine) substrate solution was added to each well, followed by incubation with shaking at 400 rpm for 10 min. The enzymatic reaction was terminated by adding 100 μL of stop solution, and absorbance was measured at 450 nm using a microplate reader (BioTek, Winooski, VT, USA).

### 2.8. Cell Culture

The human SH-SY5Y neuroblastoma cell line was cultured in Dulbecco’s Modified Eagle Medium (DMEM; Gibco, Grand Island, NY, USA) supplemented with 10% fetal bovine serum (FBS) and 1% penicillin (10,000 units/mL)/streptomycin (10,000 μg/mL) solution in a humidified incubator at 37 °C with 95% air and 5% CO_2_. Cells at a passage number less than 20 were utilized in this experiment. A total of 6 × 10^5^ cells were seeded in 6 cm dishes containing growth medium. After 24 h, the medium was replaced with DMEM containing 10 μM retinoic acid (RA) for 5 days to induce differentiation. Differentiated cells were then treated with 30 mM high glucose and 200 μM palmitic acid (GP) and AE fractions at various concentrations (a low dose of F1, 5 μg/mL; a high dose of F1, 10 μg/mL; a low dose of F2, 5 μg/mL; and a high dose of F1, 10 μg/mL) for 24 h. Cell images were captured using phase-contrast microscopy, and neurite outgrowth length was quantified using ImageJ, version 1 (NIH, Bethesda, MD, USA).

### 2.9. Immunoblot

Cells were lysed on ice using cold RIPA lysis buffer (Visal Protein, Taipei, Taiwan) supplemented with a protease inhibitor (Abcam, Cambridge, UK). Total protein concentration was determined using the Bradford protein assay (Bio-Rad Laboratories, Hercules, CA, USA). Equal amounts of protein (30 μg per sample) were separated by sodium dodecyl sulfate–polyacrylamide gel electrophoresis (SDS-PAGE) and subsequently transferred onto polyvinylidene difluoride (PVDF) membranes (Millipore, Billerica, MA, USA). The membranes were blocked with 5% skim milk and incubated overnight at 4 °C with the specified primary antibodies. Following incubation with the appropriate secondary antibodies, protein bands were detected using an enhanced chemiluminescence (ECL) reagent (Millipore, Billerica, MA, USA). Signal intensity was quantified using the ImageJ analysis system (NIH, Bethesda, MD, USA).

### 2.10. Immunocytochemistry (ICC) Analysis

After completing the treatments as aforementioned, cells were fixed with 4% cold paraformaldehyde for 10 min and permeabilized with 0.1% Triton X-100 for 5 min. Following a 30 min blocking step with 1% bovine serum albumin, cells were incubated overnight at 4 °C with primary antibodies targeting GFAP (1:200; Cat. No. CTX108711, GeneTex, Hsinchu, Taiwan), MAP2 (1:250; Cat. No. 13-1500, Invitrogen, Carlsbad, CA, USA), DCX (1:250; Cat. No. ab207175, Abcam, Cambridge, UK) or NeuN (1:200; Cat. No. ab177487, Abcam, Cambridge, UK). After three washes with PBS, cells were incubated for 1 h at room temperature with either green-fluorescent anti-rabbit IgG-488 (4 µg/mL) or red-fluorescent anti-rabbit IgG-584 (2 µg/mL). F-actin was stained using Phalloidin (eBioscience, San Diego, CA, USA). After staining, slides were mounted with ProLong ™ Diamond Antifade reagent containing DAPI (Cat. No. P36962, Invitrogen, Carlsbad, CA, USA). Fluorescence images were acquired using a BX53 fluorescence microscope (Olympus, Tokyo, Japan).

### 2.11. Statistical Analysis

Statistical analyses were conducted using Graph Pad Prism version 10 (San Diego, CA, USA). The results from animal experiments were displayed as mean  ±  standard error (SE). A one-way analysis of variance (ANOVA) was used to test the significant effect of STZ + HFD and AE on parameters with a post hoc analysis of the Holm–Sidak test. The results from the cell experiments were presented as the mean  ±  standard deviation (SD) from three independent experiments. Comparisons between groups were performed using Student’s *t*-test or a one-way analysis of variance (ANOVA) followed by Duncan’s multiple range test. A two-tailed *p*-value of <0.05 was considered statistically significant.

## 3. Results

### 3.1. Effect of AE Fractions on Blood Profiles

Table 1 presents the blood profiles observed at the conclusion of the experiment. In the NC group, ApoE^−/−^ mice exhibited an approximately 8-fold increase in total cholesterol levels. Induction with STZ and HFD (STZ + HFD group) resulted in an even greater elevation in cholesterol levels. However, treatment with fraction F2 significantly reduced total cholesterol levels in the STZ + HFD group (1545.50 vs. 1398.67 mg/dL). A similar trend was observed for TG and HDL-C. Both AE fractions effectively reduced serum TG levels, while F1 significantly lowered HDL-C levels in the STZ + HFD group. Additionally, ApoE^−/−^ mice (NC group) demonstrated a 10-fold increase in LDL-C, with the STZ + HFD group exhibiting more than a 200% increase compared to the control group. Treatment with AE fractions significantly reduced LDL-C levels. The ratio of LDL to HDL was represented as the atherogenic index [37,38], which was significantly increased to 11-fold in the STZ + HFD group compared to the control group. It was reduced by treatment with F1 or F2, but the results were not statistically significant. Notably, serum glucose and HbA1c levels remained unchanged in the NC group but were significantly elevated in the STZ + HFD group. Treatment with AE fractions effectively reduced serum glucose and HbA1c levels.

### 3.2. AE Fractions Enhance Cognitive Function in STZ + HFD-Treated ApoE^−/−^ Mice

Cognitive performance was assessed using the spontaneous alternation test (Figure 1B). While the alternation ratio in the NC group did not differ significantly from that of the control group, the STZ + HFD group exhibited a noticeable decline by 8% as early as week 4. By the end of the experiment, STZ + HFD administration resulted in significant memory impairment, as demonstrated by an 18% reduction in the alternation number, a 49% decrease in total arm entries, and a 36% decline in spontaneous alternation percentage compared to the NC group. Notably, treatment with the AE fraction, particularly F1, effectively mitigated these STZ + HFD-induced deficits in spatial memory (Figure 1D–F). In the spatial recognition test (Figure 1C), the STZ + HFD group exhibited a decreased percentage of entries into the novel arm, with reductions of 10% at week 4 and 23% at week 8 relative to the NC group (Figure 1G). The administration of AE fractions significantly reversed these deficits, suggesting improved spatial recognition ability. Further analyses revealed that the STZ + HFD group exhibited significant reductions in both the distance traveled and the time spent in the novel arm at weeks 4 and 8 (Figure 1H,I). These impairments were substantially restored following treatment with fraction F1. Although treatment with fraction F2 showed a trend toward improvement, the effects did not reach statistical significance. These findings suggest that AE fractions may enhance memory and exploratory behavior.

### 3.3. AE Fractions Mitigate Hippocampal Degeneration in STZ + HFD-Treated ApoE^−/−^ Mice

**Figure 2:**
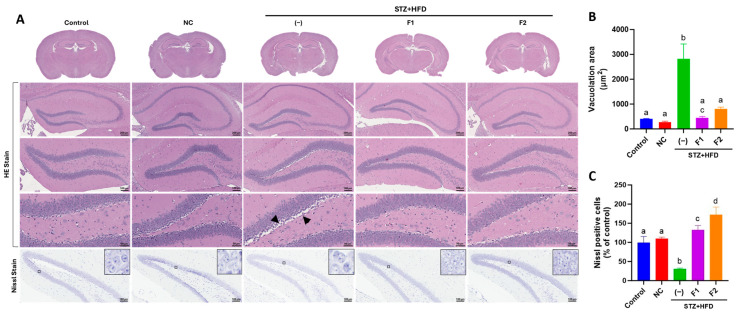
The effect of AE fractions on the histological alterations of the hippocampal DG. (**A**) H&E and Nissl staining revealed histological changes in the DG of the hippocampus. The black arrow head points to the vacuolation site. (**B**) A quantitative assessment of vacuolization based on H&E-stained sections and (**C**) a quantitative analysis of Nissl-stained neurons (blue-purple color). Values are represented as mean ± SE; values not sharing a common letter are significantly different (*p* < 0.05).

The HE staining of the hippocampal dentate gyrus (DG) revealed distinct histological differences between the NC and STZ + HFD groups. In the STZ + HFD group, marked vacuolation was observed, indicative of neuronal degeneration. Nissl staining, which identifies neurons by the presence of Nissl substance stained blue-purple, showed a diffuse and diminished staining pattern in the STZ + HFD group. This reduction in staining intensity suggests the potential disruption of the nuclear membrane (Figure 2A). Quantitative histological analysis revealed a significant reduction in vacuolization in the hippocampal DG of the STZ + HFD group treated with AE fractions. Specifically, fractions F1 and F2 reduced vacuolization by 84% and 71%, respectively, compared to the untreated STZ + HFD group (Figure 2B). In parallel, Nissl stain intensity within the hippocampal DG was markedly increased to 4.3-fold in the F1-treated group and 5.5-fold in the F2-treated group compared with the STZ + HFD group (Figure 2C), suggesting pronounced neuroprotective effects of both fractions.

### 3.4. AE Fractions Promote Hippocampal Differentiation and Reduce Oxidative Stress

**Figure 3:**
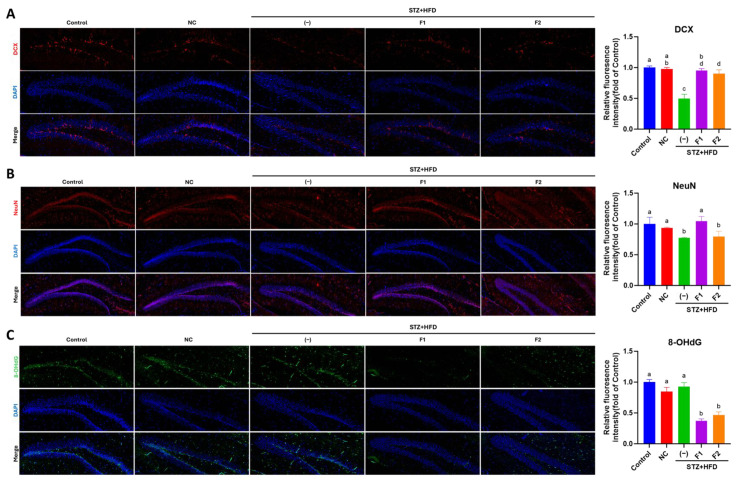
AE fractions enhanced hippocampal neurogenesis and attenuate oxidative damage. Immunofluorescence was conducted to determine the expression levels of (**A**), DCX; (**B**), NeuN; (**C**), 8-OHdG in the hippocampal DG. The quantification data are presented in the right panels, respectively. Values are represented as mean ± SE; values not sharing a common letter are significantly different (*p* < 0.05).

The results of the immunofluorescence analysis are shown in Figure 3. Compared to the control and NC groups, the STZ + HFD group exhibited a marked reduction in DCX expression in the hippocampal DG, with levels decreased to approximately 50% of that observed in the control group. Treatment with AE-derived fractions F1 and F2 significantly increased DCX expression (Figure 3A). A similar trend was observed for NeuN, a marker of mature neurons, where its expression was significantly reduced in the STZ + HFD group. Notably, treatment with F1 significantly restored NeuN expression, while F2 did not exert a comparable effect (Figure 3B). These findings suggest that AE fractions may promote neuronal differentiation during development. In terms of oxidative stress, although the 8-OHdG levels were not elevated in STZ + HFD group compared to the NC group, treatment with fractions F1 and F2 significantly reduced 8-OHdG levels, with reductions of approximately 60% and 50%, respectively, compared to the untreated STZ + HFD group. These findings indicate that AE fractions are quite effective in mitigating oxidative stress (Figure 3C).

### 3.5. AE Fractions Increase Blood BDNF Levels

**Figure 4:**
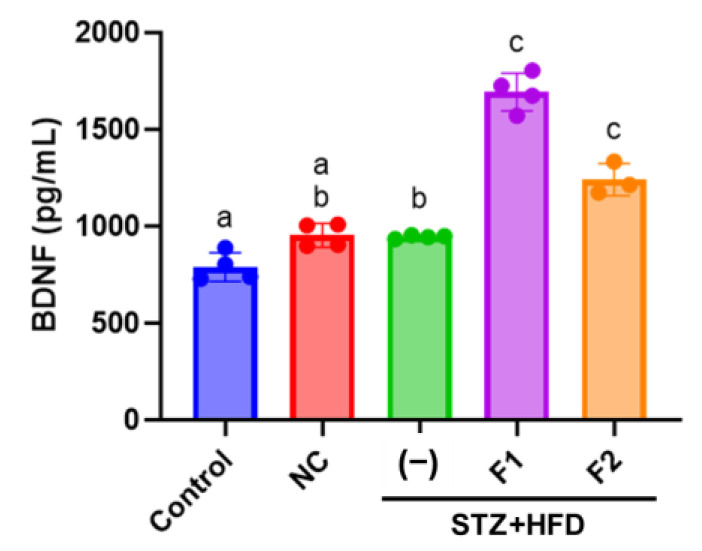
AE fractions increased serum BDNF levels in HFD/STZ-treated ApoE^−/−^ mice. The concentration of BDNF in blood samples was determined by ELISA. Values are represented as mean ± SE; values not sharing a common letter are significantly different (*p* < 0.05).

BDNF levels remained unchanged, with comparable expression observed across the control, NC, and STZ + HFD groups. Treatment with AE fractions further enhanced blood BDNF levels, with fraction F1 increasing BDNF expression to 1.8-fold relative to the control (Figure 4).

### 3.6. AE Fractions Promote Neurite Growth and Neuronal Differentiation in Differentiated SH-SY5Y Cells

**Figure 5:**
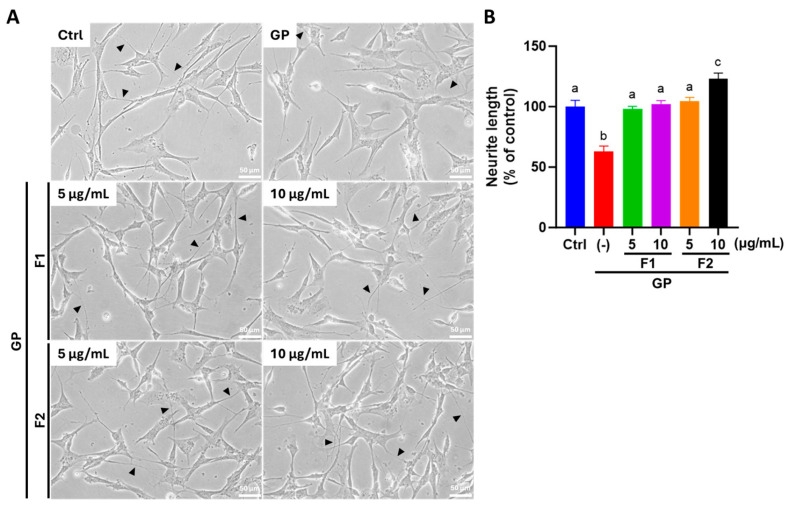
AE fractions promoted neurite outgrowth in differentiated SH-SY5Y cells treated with glucose and PA. Ctrl (control), cells were treated with 10 μM of RA for 5 days to induce differentiation. GP, cells were differentiated with 10 μM of RA for 5 days, followed by incubation with 30 mM glucose and 200 μM PA. Cells were subsequently treated with varying concentrations of F1 or F2 for 24 h. The concentration of AE fractions included 5 or 10 μg/mL of F1; 5 or 10 μg/mL of F2. (**A**) Cell morphology was documented via phase-contrast microscopy at 200× magnification, scale bar: 50 μm. The black arrow heads indicated the neurite; (**B**) neurite length was quantified using ImageJ. Values from three independent experiments are represented as mean ± SD; values not sharing a common letter are significantly different (*p* < 0.05).

**Figure 6:**
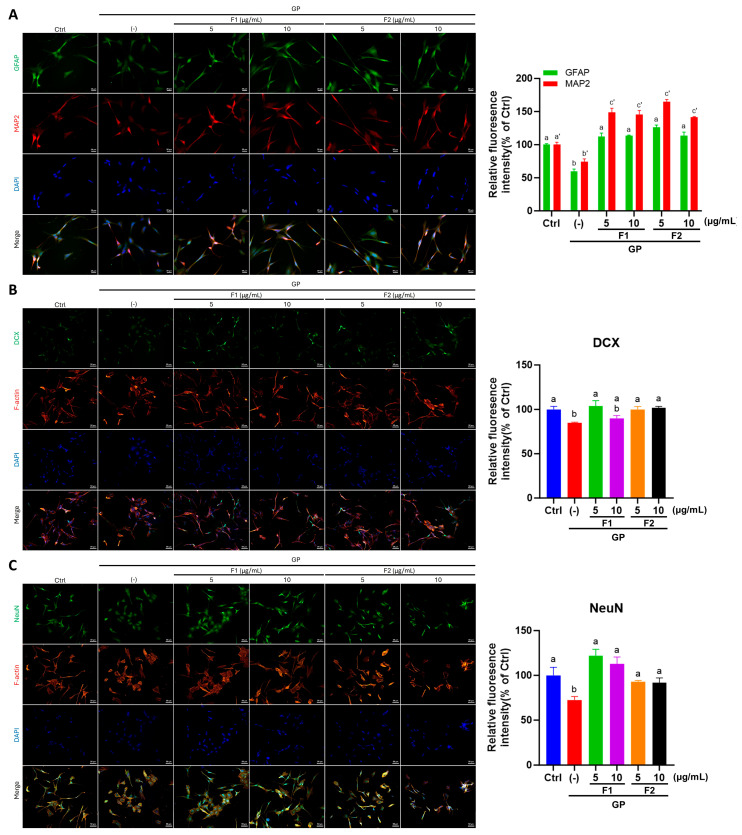
AE fractions on enhanced neuronal in differentiated SH-SY5Y cells treated with glucose and PA. (**A**) ICC staining demonstrated the co-localization of GFAP (green) and MAP2 (red) within DAPI-stained nuclei (blue). (**B**) ICC analysis revealed the spatial distribution of DCX (green) and F-actin (visualized with Phalloidin, red) relative to DAPI-labeled nuclei (blue). (**C**) The coimmunostaining of NeuN (green) and F-actin (red) was performed, with DAPI counterstaining to visualize nuclei (blue). Scale bar: 20 μm. A quantitative analysis of these observations is presented in the right panels, respectively. Values from three independent experiments are represented as mean ± SD; values not sharing a common letter are significantly different (*p* < 0.05). a′, b′, and c′ in the panel in the upper right corner present the quantitative analysis of MAP2 expression.

As shown in Figure 5A, SH-SY5Y cells exposed to high glucose and palmitate (GP) exhibited a loss of dendritic processes and cell connections. However, treatment with AE fractions markedly restored normal cell morphology. As shown in Figure 5B, a quantitative analysis of neurite length revealed that AE fractions, F1 and F2, in varying concentrations, effectively promoted neurite outgrowth, with fraction F2 increasing neurite length beyond the control levels. A further analysis of neuronal differentiation markers revealed that the expression levels of GFAP and MAP2 were significantly reduced in the GP group by 41% and 26%, respectively. These reductions were significantly reversed following treatment with AE fractions (Figure 6A), indicating a restorative effect on neuronal differentiation. Similarly, the immunofluorescence detection of DCX and NeuN exhibited the same trend, further supporting the role of AE fractions in enhancing neuronal differentiation (Figure 6B,C).

### 3.7. Effect of AE Fractions on Neuronal Oxidative Stress and BDNF Signaling

**Figure 7:**
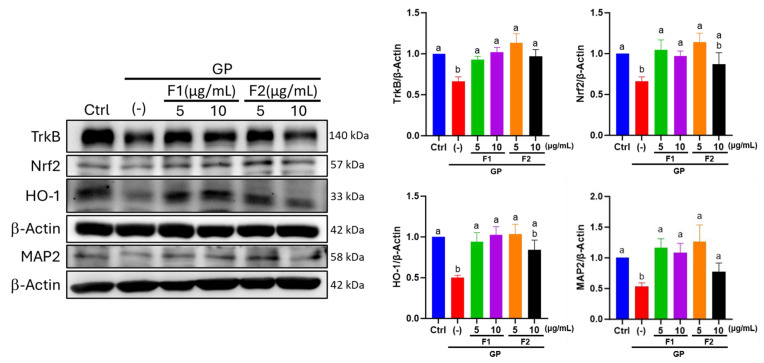
Effects of AE fractions on the proteins related to oxidative stress or BDNF signaling in differentiated SH-SY5Y cells treated with glucose and PA. Total protein was extracted from cell lysates and analyzed via immunoblotting. β-Actin served as the loading control. The relative protein level from three independent experiments shown as fold mean ± SD are quantified by a densitometric analysis of immunoblot bands. Values not sharing a common letter are significantly different (*p* < 0.05).

Nuclear factor-erythroid 2-related factor 2 (Nrf2) and heme oxygenase-1 (HO-1) are key protective markers activated in response to oxidative stress. In the GP group, the levels of Nrf2 and HO-1 were significantly reduced to 0.66- and 0.50-fold, respectively, compared with the control group. However, treatment with 5, 10 μg/mL of F1 or 5 μg/mL of F2 effectively restored their expression, whereas 10 μg/mL of F2 exhibited limited efficacy in this regard. Similarly, TrkB, a critical component of BDNF signaling, was downregulated to 0.66-fold in the GP group compared to the control group, whereas it significantly increased following treatment with both AE fractions. This suggests that AE fractions may facilitate the recovery of BDNF signaling. Importantly, the immunoblot analysis confirmed the immunocytochemical findings related to MAP2 protein expression, thereby providing additional evidence for the neuroprotective effects of the AE fractions (Figure 7).

## 4. Discussion

In this study, we demonstrate that the combination of STZ and HFD induces a CVD model in ApoE^−/−^ mice, leading to hyperlipidemia, hyperglycemia, cognitive and memory impairment, and hippocampal damage. Our findings indicate that treatment with AE improves lipid profiles and alleviates hyperglycemia while mitigating hippocampal neuronal damage. AE fractions reduce oxidative stress markers, including 8-OHdG, and modulate NeuN and DCX levels, which are associated with early and late stages of neuronal development, respectively. These effects contribute to enhanced learning and memory abilities. Furthermore, in a neuroblastoma cell model where RA-induced differentiation generates neuroblast-like cells, exposure to high glucose and palmitate was used to simulate a hyperglycemic and hyperlipidemic environment. Under these conditions, AE was found to promote neurite outgrowth and neuronal differentiation, accompanied by the increased expression of HO-1, Nrf2, and BDNF. Notably, F1 exhibited superior antioxidant properties, whereas F2 demonstrated greater efficacy in promoting neurite growth.

The neuroprotective effects of AE have been documented in various experimental models [26,39,40]. In SH-SY5Y cells expressing the H63D variant linked to AD risk, AE ethanol extract reduced oxidative stress, potentially preventing the initiation of neurodegeneration [41]. Beyond its neuroprotective effects, AE has also demonstrated cardiovascular protective properties in both clinical and animal studies [42,43,44]. In our current study, in the in vivo study, AE mitigated hippocampal neuronal lesions by reducing oxidative stress, promoting neuronal development through the increased expression of NeuN and DCX, and elevating blood BDNF levels in a CVD model. In the in vitro study, AE was shown to promote neurite extension and upregulate the expression of DCX and NeuN in high glucose- and palmitate-treated differentiated SH-SY5Y cells, thereby enhancing neural cell differentiation. Additionally, AE reduces 8-OHdG and enhances BDNF signaling. These findings not only emphasize AE in preserving the structural integrity of the nervous system but also suggest its potential in mitigating the risk of CVD progression to vascular dementia. AE shows protective effects on both the nervous and cardiovascular systems, highlighting its potential therapeutic value in health maintenance and disease prevention.

Hyperlipidemia or dyslipidemia is a significant risk factor for cardiovascular disease, hypertension, and cerebrovascular stroke and may, therefore, be associated with vascular dementia. A longitudinal cohort study conducted by Pan et al. demonstrated that an earlier age at the diagnosis of hyperlipidemia was correlated with an increased risk of subsequent dementia [45]. In a hyperlipidemic mouse model, elevated levels of total cholesterol and triglycerides with co-occurred aortic atherosclerosis were observed [46]. In addition, reduced cerebral blood flow, impaired remote memory, and cerebral vascular dysfunction were documented in hyperlipidemic mice [46]. AE has been demonstrated to reduce serum total cholesterol, low-density lipoprotein cholesterol, and glucose levels [19,47,48]. A systematic review and meta-analysis have demonstrated that AE consumption significantly improves blood glucose, HbA1c, TG, and TC levels in adults [42]. A study suggests that the cardioprotective effects of AE may be mediated through its lipid-lowering properties [48]. ApoE^−/−^ mice are known to exhibit delayed lipoprotein clearance, which contributes to the development of hyperlipidemia or dyslipidemia and ultimately leads to atherosclerosis [49], resembling human pathological conditions [50,51]. In the present study, ApoE^−/−^ mice co-treated with an HFD and STZ exhibited hyperlipidemia, an increased atherogenic index, and cognitive decline. The ability of AE to reduce blood lipid levels may play a crucial role in lowering the atherogenic index, slowing the progression of hippocampal lesions, and ultimately mitigating the risk of vascular dementia.

Endogenous ApoE deficiency leads to an imbalance in cholesterol loading within macrophages, which subsequently stimulates cytokine release and protease secretion, thereby triggering inflammation and oxidative stress [52]. AE has been reported to modulate oxidative stress markers [53]. In an amyloid-beta (Aβ)-injected AD-like model, AE-derived flavonoids demonstrated efficacy in ameliorating cognitive deficits. This effect is hypothesized to be mediated by the regulation of BDNF expression in the cortex and hippocampus, the activation of CREB/ERK and PI3K/AKT/GSK3β signaling pathways, and the attenuation of neuro-oxidative stress and inflammation [54]. BDNF is synthesized not only in neurons and glial cells but also in various peripheral tissues [55]. Under stress conditions, such as the early stages of neurodegeneration seen in Alzheimer’s disease (AD), serum BDNF levels may increase as a compensatory mechanism to mitigate neurodegeneration [56]. As a result, circulating BDNF levels are subject to multiple confounding influences, which may explain the absence of a significant reduction in BDNF levels in the STZ + HFD group compared to the control group. TrkB is a receptor that directs BDNF-related signaling activity; a significant decrease occurred in TrkB protein levels following treatment with high glucose and palmitate (Figure 7). These findings suggest that, although serum BDNF levels were not significantly altered in STZ + HFD-treated animals, the expression or functionality of its primary receptor, TrkB, may be suppressed. This suppression could impair downstream signaling pathways and potentially attenuate the neuroprotective and neurorestorative effects mediated by BDNF.

Previous studies have demonstrated that AE reverses cognitive deficits and attenuates dexamethasone-induced morphological alterations in the hippocampal CA3 region of mice [39]. In the present study, ApoE^−/−^ mice subjected to a combined STZ and HFD regimen exhibited 8-OHdG in the hippocampal DG region, indicative of oxidative DNA damage. AE treatment significantly attenuated this oxidative damage. These findings suggest that the observed mitigation of cognitive decline by AE may be attributed, at least in part, to its antioxidative properties. Notably, in the photos of the STZ + HFD group, 8-OHdG expression was also observed in hippocampal regions outside the DG and appeared higher than that in the control and NC groups. Given that STZ + HFD treatment induces cognitive disorders, the elevated expression of 8-OHdG in non-DG hippocampal regions may be associated with cognitive impairment and warrants further investigation. On the other hand, previous studies have demonstrated that treatment with F1 or F2 alone in 7-week-old Sprague-Dawley (SD) rats for 12 weeks did not result in significant differences in serum thiobarbituric acid reactive substance (TBARS) levels compared to the normal control group [57]. These findings suggest that F1 or F2 treatment alone does not significantly influence oxidative stress. However, given the distinct metabolic profile of ApoE^−/−^ mice compared to SD rats, further investigation is required to determine whether F1 or F2 treatment without STZ + HFD induces changes in oxidative stress in the ApoE^−/−^ mouse model.

Nrf2 is a transcription factor that regulates the expression of antioxidant and anti-inflammatory molecules in mammalian cells [58]. HO-1, a gene transcriptionally regulated by Nrf2, is highly inducible by nitric oxide, heavy metals, growth factors, cytokines, and modified lipids [59]. The disruption of the Nrf2/HO-1 signaling pathway has been implicated in the pathogenesis of age-related disorders including neurodegeneration [58]. Our in vitro findings and in vivo studies demonstrated that AE reduces 8-OHdG generation and enhances the expression of antioxidant molecules. Notably, AE treatment, particularly the F1 fraction, exhibited efficacy across a dose range. As previously reported, fraction F1 is predominantly composed of quercetin glucosides and triterpene esters (4.301 mg/g dry weight) [33], both of which exhibit antioxidant and lipid-lowering activities. Quercetin has been shown to protect the cerebral cortex of rats against ethanol-induced oxidative damage by upregulating the mRNA and protein levels of Nrf2 and HO-1 [60]. Quercetin inhibits neuronal degeneration by increasing the number of intact neurons and acts as an anti-inflammatory agent by decreasing the production of the cytokine interleukin-1β [60]. The high dietary intake of quercetin has been associated with decreased circulating levels of LDL, thus managing obesity and metabolic syndrome [61]. Despite these beneficial effects, the oral bioavailability of quercetin remains relatively low, which may limit its clinical efficacy. Quercetin in plants predominantly exists in glycosidic forms. Upon ingestion, a portion of these quercetin glycosides undergoes glucuronide or sulfate conjugation and is subsequently absorbed in the small intestine. The resulting conjugated metabolites produced via the activity of plasma β-glucuronidase function as food-derived anti-atherogenic agents. A significant proportion of quercetin glycosides subsequently transit to the large intestine, where they undergo microbial catabolism. This process yields deglycosylated aglycones and chain scission metabolites [21]. These microbial derivatives are subsequently absorbed into the systemic circulation, potentially exerting beneficial effects on vascular function. Epidemiological studies, including human cohort and intervention trials, have consistently demonstrated an inverse association between the consumption of quercetin glycoside-rich foods, notably onions, and the incidence of CVD [62]. The quercetin glucoside in AE (4.901 mg/g dry weight) is notably higher than that in onion (1.8 mg/100 g). This improved bioavailability has been linked to a greater reduction in body fat, enhancement of endothelial function, and attenuation of cognitive decline [63]. In current cardiovascular disease models, the observed attenuation of cognitive decline may be attributed to the reduction in cardiovascular risk. Accordingly, the extraction of quercetin glycosides from edible food sources presents a promising and practical approach for functional food and nutraceutical development; optimizing extraction techniques to preserve glycosidic structures as biologically active linkers should be a key consideration in future research and applications. Additionally, triterpene fractions extracted from *Centella asiatica* have been reported to exert anti-neuroinflammatory and antioxidant effects through regulating the Nrf2/HO-1 signaling pathway [64]. In line with current findings, the observed effects of the F1 fraction may be attributed to the combined activity of quercetin glycosides and triterpenes. Beyond enhancing bioavailability, the synergistic interactions among the constituent compounds in natural product extracts may facilitate realizing their biological functions.

Intriguingly, our current in vitro findings demonstrate that, while the F2 fraction consistently stimulates microtubule-associated protein 2 (MAP2) expression and neurite outgrowth, high doses of F2 appear to exhibit limited efficacy in enhancing Nrf2/HO-1 signaling. As previously reported, F2 is predominantly composed of high-molecular-weight polysaccharides. Considering its in vivo activity, F2 may facilitate a distinct transport mechanism from the gastrointestinal tract to the brain, thereby promoting neuronal differentiation, improving cognition, and attenuating oxidative stress. Previous studies have suggested that AE increases short-chain fatty acid production by modulating the composition of the gut microbiota [65]. Our prior research also indicates that F2 improves hippocampal function, potentially through alterations in gut microbiota composition [26]. Therefore, the gut–brain axis may play a significant role in the in vivo effects of F2. However, the observed limited induction of antioxidant molecules by high doses of F2 in vitro suggests a potential paradox. It is speculated that this discrepancy may arise from high concentrations of polysaccharides physically obstructing cell surface receptors, thereby impeding signaling pathways specifically associated with antioxidant responses.

The present study has several limitations that warrant further investigation. First, 7-week-old ApoE^−/−^ mice were used as the animal model. Although AE attenuated hippocampal DG damage under HFD + STZ induction, accompanied by increased NeuN and DCX levels, young animals retain the capacity for neuronal growth and differentiation. Consequently, the use of 7-week-old mice may not adequately reflect aging conditions, thereby limiting the ability in this study to elucidate the relationship between neurodegeneration and aging. Future research on the neuroprotective effects of AE should consider employing naturally aged animal models to enhance translational relevance. Second, the functional components of F1, including quercetin glucoside and triterpene ester, may exert antioxidant effects that help attenuate HFD- and STZ-induced damage in the hippocampal DG. In contrast, F2, which consists primarily of high-molecular-weight polysaccharides, is hypothesized to improve hippocampal function through the modulation of gut microbiota composition; however, this mechanism requires further experimental validation. Third, epidemiological and clinical evidence has established that patients with CVD are at an increased risk of developing vascular cognitive impairment. However, cognitive disorders are progressive in nature, and identifying key biomarkers in CVD patients may represent an effective strategy for the early prevention of vascular cognitive impairment. Fourth, the specific stage at which cognitive decline begins during the progression from dyslipidemia to CVD remains unclear. Furthermore, the potential biomarkers for monitoring the onset of cognitive impairment and their correlation with disease progression require further investigation. This study employed an animal model to investigate the potential of AE in attenuating cognitive disorders under CVD conditions. However, whether AE exhibits similar effects in humans remains to be validated through further investigation.

## 5. Conclusions

In summary (graphic abstract), AE mitigates oxidative stress, promotes neuron differentiation and development, and improves the hippocampal function of memory and exploring. AE fractions F1 and F2 have their own strengths, respectively, and demonstrate promise for use as adjuvants to prevent dyslipidemia- and hyperglycemia-induced cognition decline. AE might potentially affect the human hippocampus to improve cognitive function; the precise mechanism of the neuroprotective effect of these plant extracts should be further investigated.

## Figures and Tables

**Table 1:** Effects of AE on body weight, plasma lipid, and glucose in ApoE^−/−^ mice exposed to STZ + HFD ^1^.

	Control	NC	STZ + HFD	STZ + HFD + F1	STZ + HFD + F2 ^2^
Body weight (g)	26.45 ± 0.25 ^a^	28.53 ± 1.08 ^a^	23.53 ± 1.09 ^b^	24.13 ± 0.38 ^b^	25.83 ± 1.58 ^a^
HbA1c (%)	4.03 ± 0.10 ^a^	3.93 ± 0.10 ^a^	7.00 ± 0.36 ^b^	4.95 ± 0.88 ^a^	5.00 ± 0.35 ^a^
Glucose (mg/dL)	287.75 ± 58.39 ^a^	202.50 ± 24.14 ^ab^	454.50 ± 54.88 ^ac^	220.75 ± 40.14 ^ab^	193.67 ± 62.22 ^ab^
Cholesterol (mg/dL)	105.75 ± 4.03 ^a^	807.25 ± 77.68 ^b^	1545.50 ± 87.58 ^c^	1415.00 ± 51.68 ^c^	1398.67 ± 45.83 ^d^
TG (mg/dL)	65.75 ± 6.95 ^a^	92.25 ± 7.76 ^a^	163.25 ± 25.10 ^b^	88.00 ± 18.24 ^a^	85.67 ± 11.06 ^a^
HDL-c (mg/dL)	59.75 ± 6.13 ^a^	100.50 ± 12.66 ^b^	135.25 ± 30.61 ^c^	100.75 ± 15.31 ^b^	109.33 ± 13.61 ^bc^
LDL-c (mg/dL)	10.00 ± 1.63 ^a^	109.75 ± 10.53 ^b^	244.75 ± 35.49 ^c^	132.75 ± 24.58 ^b^	146.00 ± 29.72 ^b^
LDL/HDL Ratio	0.17 ± 0.03 ^a^	1.10 ± 0.15 ^b^	1.89 ± 0.56 ^c^	1.32 ± 0.20 ^bc^	1.33 ± 0.14 ^bc^

^1^ ApoE^−/−^ mice were treated with intraperitoneal-injected STZ at a dose of 50 mg/kg body weight and an HFD and subsequently supplemented with 0.65 mg/kg body weight of F1 or F2 for 8 weeks. The control and NC (ApoE^−/−^ mice) groups were given the control diet. ^2^ Each value is expressed as mean ± SE (n = 3 or 4). The results were statistically analyzed using ANOVA. Values not sharing a common letter in the same row are significantly different (*p* < 0.05).

## Data Availability

The data used to support the findings of the study are available upon reasonable request from the corresponding author.

## Supplementary Materials

The following supporting information can be downloaded at https://www.mdpi.com/article/10.3390/antiox14080955/s1, Figure S1: The procedures for extracting AE fractions; Figure S2: HPLC/UV chromatograms of F1; Figure S3: Cognitive impairment in ApoE^−/−^ mice exposed to STZ + HFD. Table S1: Retention time, UV-Vis and mass spectral characteristics of the identified compounds in F1 of AE; Table S2: Monosaccharides in F2 of AE. Table S3: Plasma Hb1c, glucose, and lipid in ApoE^−/−^ mice exposed to STZ + HFD.
