# Supplementary material for: Abelmoschus esculentus Ameliorates Cognitive Impairment in Hyperlipidemic ApoE−/− Mice via Modulation of Oxidative Stress and Neuronal Differentiation
Source: Antioxidants (Basel). 2025 Aug 4;14(8):955. doi: 10.3390/antiox14080955 (PMC12383169; doi:10.3390/antiox14080955)
Supplement: Supplementary file 1 [file antioxidants-14-00955-s001.zip › antioxidants-3677401-supplementary.pdf]

Supplementary Data

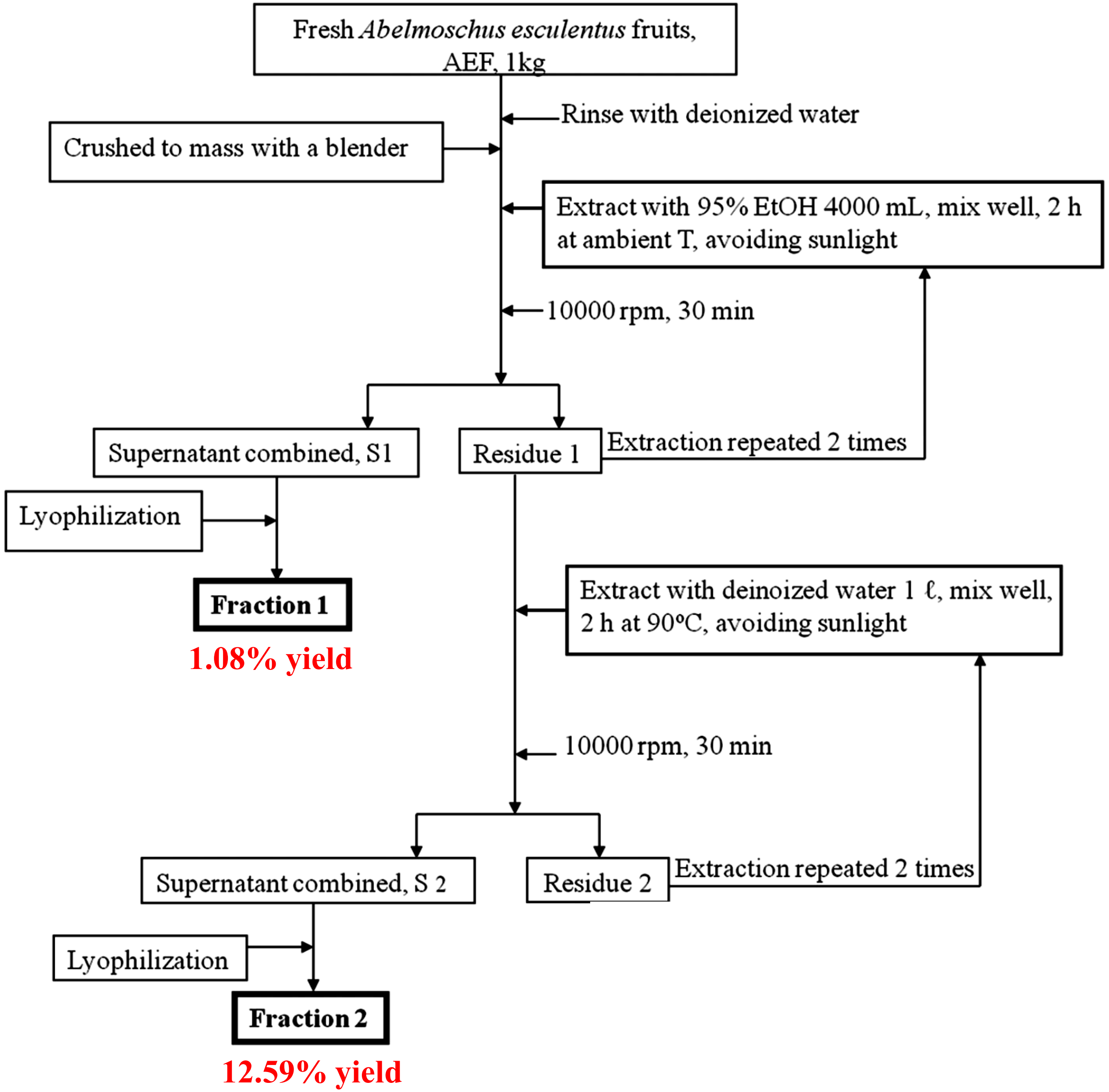

Figure S1. The procedures for extracting AE fractions.

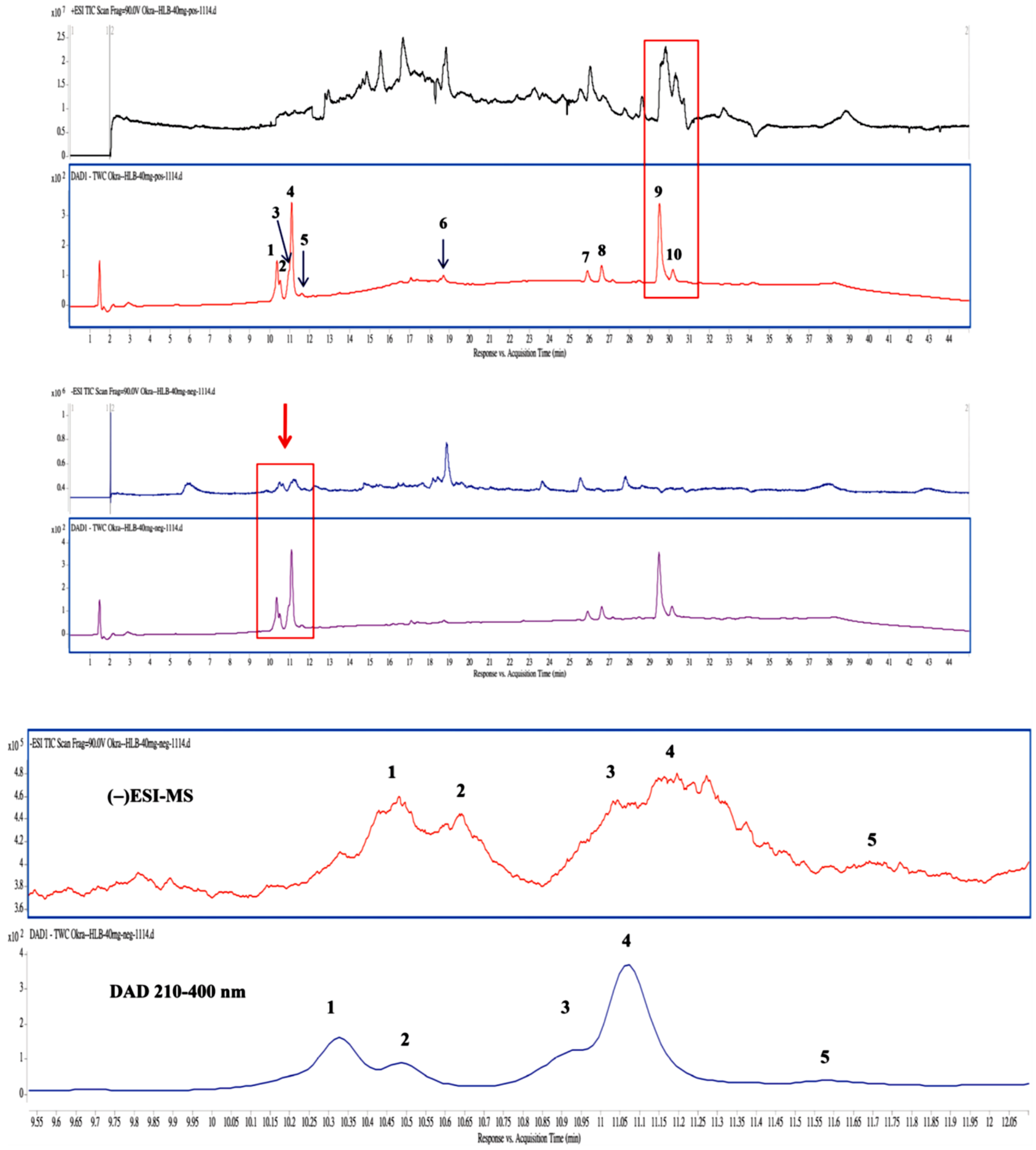

Figure S2. HPLC/UV chromatograms of F1. The absorption spectra of eluted compounds were scanned within 210–400 nm. For peak numbers refer to Table 1.

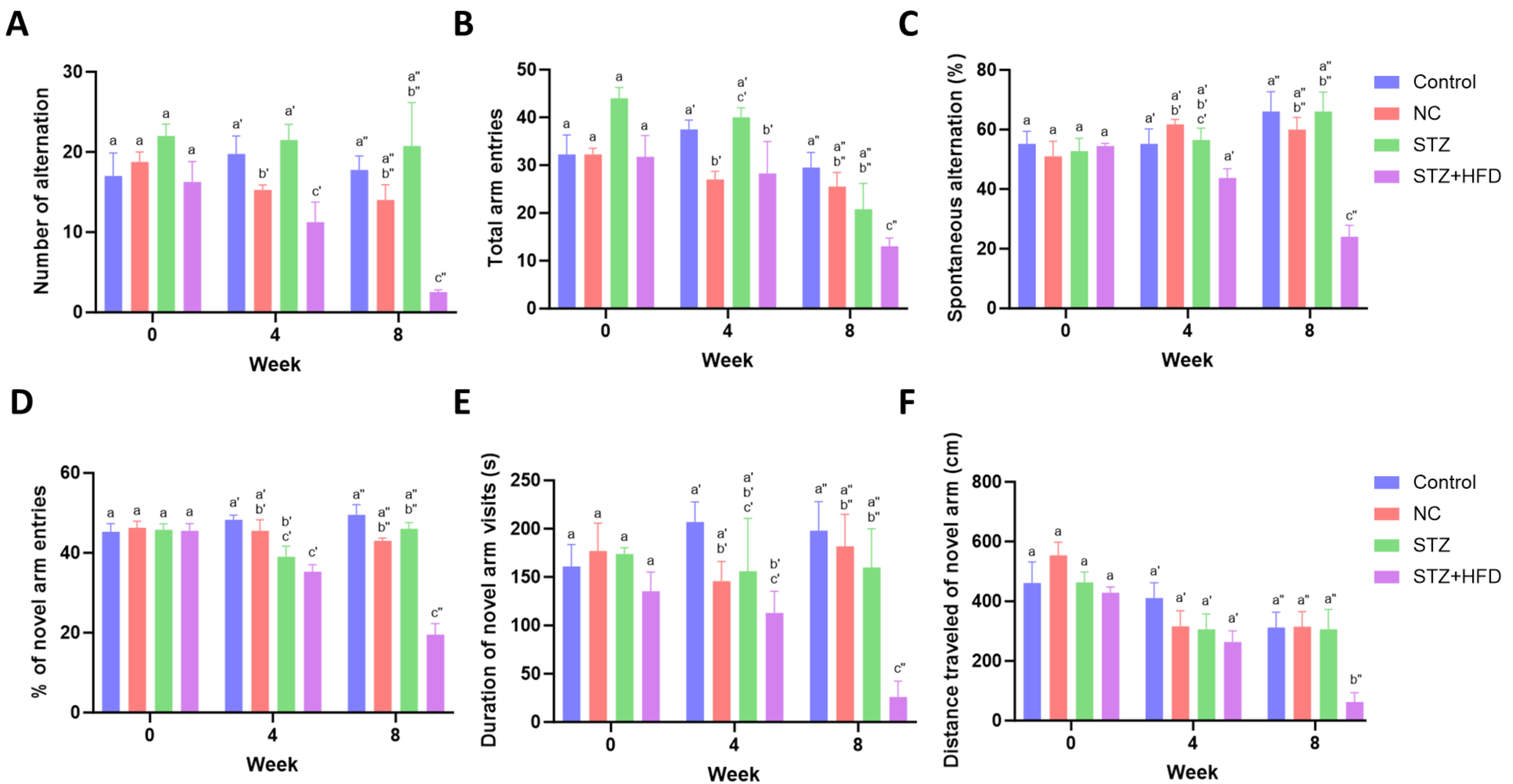

Figure S3. Cognitive impairment in ApoE$^{-/-}$ mice exposed to STZ+HFD. Cognitive performance was assessed by analyzing A, the number of alternation; B, total arm entries; C, percentage of spontaneous alternation in the Y-maze. Spatial recognition learning was assessed by measuring D, the percentage of novel arm entries; E, duration of novel arm visits; F, distance traveled of novel arm. Values were represented as mean ± SE; values not sharing a common letter are significantly different ($p < 0.05$). Compared with the ApoE$^{-/-}$ mice, no significant differences were observed in behavioral assessments in STZ-treated ApoE$^{-/-}$ mice. STZ combined with HFD to treat ApoE$^{-/-}$ mice can induce cognitive deficits significantly.

Table S1. Retention time, UV-Vis and mass spectral characteristics of the identified compounds in F1 of AE

Peak no.	Compound	t_R(min)	λ_{max}(nm)	$[M + H]^+$	$[M - H]^-$	MS^2
1	Quercetin 3-O-glucosyl (1 → 6)glucoside[a]	10.34	255, 354	ND	625	301, 300
2	Quercetin 3-O-xylosyl (1 → 2) glucoside[a]	10.50	255, 355	ND	595	300, 301, 271, 463
3	Quercetin 3-O-glucoside[a]	10.90	254, 355	ND	463	301, 300
4	Flavonoid glucoside	11.07	253, 354	ND	629	
5	Flavonoid glucoside	11.58	255, 350	ND	549	
6	Unknown	18.67	230	ND	277	134, 121, 127, 147
7	Unknown	25.84	232	593	ND	
8	Unknown	26.58	232	635	ND	
9	Pentacyclic triterpene ester[b]	29.47	232, 325, 400	621	ND	
10	Pentacyclic triterpene ester[b]	30.14	232, 323, 400	621	ND	

[a] Compounds 1, 2 and 3 were tentatively identified according to mass spectra and the matched data from Shui, et al., 2004, J. Chromatogr., A, 1048, 17–24.
[b] Compounds 9 and 10 were tentatively identified according to mass spectra and the matched data from Yun, et al., 1999, J. Nat. Prod., 62, 764–766.

Table S2. Monosaccharides in F2 of AE

Sugar	Wt%, w/w
Rhamnose	9.79
Fucose	7.30
Mannose	0.43
Galactose	18.92
Glucose	18.26
Myo-inositol	14.21
Glucosamine	8.86
Uronic acid (galacturonic acid 16.26%)	23.14

Table S3. Plasma Hb1c, glucose, and lipid in $ApoE^{-/-}$ mice exposed to STZ+HFD [1].

	Control	NC	STZ	STZ+HFD
**HbA1c (%)**	4.03 ± 0.10^{a}	3.93 ± 0.10^{ab}	6.45 ± 1.65^{c}	7.00 ± 0.36^{c}
**Glucose (mg/dL)**	287.75 ± 58.39^{a}	202.50 ± 24.14^{ab}	358.00 ± 44.95^{a}	454.50 ± 54.88^{a}
**Cholesterol (mg/dL)**	105.75 ± 4.03^{a}	807.25 ± 77.68^{b}	$1{,}384.00 \pm 84.90^{c}$	$1{,}545.50 \pm 87.58^{d}$
**TG (mg/dL)**	65.75 ± 6.95^{a}	92.25 ± 7.76^{b}	90.00 ± 6.32^{b}	163.25 ± 25.10^{c}
**HDL (mg/dL)**	59.75 ± 6.13^{a}	100.50 ± 12.66^{b}	109.25 ± 5.91^{b}	135.25 ± 30.61^{b}
**LDL (mg/dL)**	10.00 ± 1.63^{a}	109.75 ± 10.53^{b}	141.25 ± 25.51^{b}	244.75 ± 35.49^{c}
**LDL/HDL Ratio**	0.17 ± 0.03^{a}	1.10 ± 0.15^{b}	1.30 ± 0.29^{b}	1.89 ± 0.56^{b}

[1] $ApoE^{-/-}$ mice were treated with intraperitoneal-injected STZ at a dose of 50 mg/kg body weight for five consecutive days. One week post-injection, mice exhibiting fasting plasma glucose levels exceeding 200 mg/dL were considered diabetic and fed HFD for 8 weeks. The control and NC ($ApoE^{-/-}$ mice) groups were given the control diet. Each value is expressed as mean $\pm$ SE (n = 3). Results were statistically analyzed using ANOVA. Values not sharing a common letter in the same row are significantly different ($p < 0.05$). Compared with the $ApoE^{-}/^{-}$ mice, STZ-treated $ApoE^{-}/^{-}$ mice exhibited elevated serum cholesterol and LDL levels. STZ combined with HFD to treat $ApoE^{-}/^{-}$ mice can induce hyperlipidemia significantly.
